# Supplementary material for: A UMLS-based spell checker for natural language processing in vaccine safety
Source: BMC Med Inform Decis Mak. 2007 Feb 12;7:3. doi: 10.1186/1472-6947-7-3 (PMC1805499; doi:10.1186/1472-6947-7-3)
Supplement: Additional file 13 — RAP application directory. Sets up directory for RAP (RDF application for PHP) source code [file 1472-6947-7-3-S13.gz › rap/doc/rdql_grammar.htm]

BNF for RAP's RDQL engine


### RDF API for PHP V0.9.1

# BNF for RAP’s RDQL engine

**NON-Terminals**

|  |  |  |
| --- | --- | --- |
| CompilationUnit | ::= | Query |
| CommaOpt | ::= | (“**,**”)? |
| Query | ::= | SelectClause  (SourceClause)?  TriplePatternClause  (ConstraintClause)?   (PrefixesClause)? |
| SelectClause | ::= | (“**SELECT**” Var (CommaOpt Var)\* \| “**SELECT \***") |
| SourceClause | ::= | (“**FROM**” \| “**SOURCE**”) QuotedURI |
| TriplePatternClause | ::= | “**WHERE**” TriplePattern (CommaOpt TriplePattern)\* |
| TriplePattern | ::= | “**(**“ VarOrURI CommaOpt VarOrURICommaOpt VarOrUriOrLiteral “**)**” |
| ConstraintClause | ::= | “**AND**” Expression (“**,**” Expression)\* |
| PrefixesClause | ::= | “**USING**” PrefixDecl (CommaOpt PrefixDecl)\* |
| PrefixDecl | ::= | Identifier “**FOR**” QuotedURI |
| Expression | ::= | ConditionalOrExpression |
| ConditionalOrExpression | ::= | ConditionalXorExpression (“**\|\|**” ConditionalXorExpression)\* |
| ConditionalXorExpression | ::= | ConditionalAndExpression |
| ConditionalAndExpression | ::= | ValueLogical (“**&&**” ValueLogical)\* |
| ValueLogical | ::= | StringEqualityExpression |
| StringEqualityExpression | ::= | EqualityExpression ((StrEqOp EqualityExpression) \| (RegexMatchOp RegexPattern)) |
| StrEqOp | ::= | “**EQ**” \| “**NE**” |
| RegexMatchOp | ::= | “**~~**” \| “**=~**” \| “**!~**” |
| RegexPattern | ::= | <REGULAR\_EXPRESSION> |
| EqualityExpression | ::= | RelationalExpression (EqOp RelationalExpression)? |
| EqOp | ::= | “**==**” \| “ **!=**” |
| RelationalExpression | ::= | AdditiveExpression (RelOp AdditiveExpression) ? |
| RelOp | ::= | “**<**” \| “**<=**” \| “**>**” \| “**>=**” |
| AdditiveExpression | ::= | MultiplicativeExpression (AddOp MultiplicativeExpression)\* |
| AddOp | ::= | “**+**” \| “**-**” |
| MultiplicativeExpression | ::= | UnaryExpression (MulOp UnaryExpression)\* |
| MulOp | ::= | “**\***” \| “**/**” \| “**%**” |
| UnaryExpression | ::= | PrimaryExpression \| “**+**” UnaryExpression \| “**-**” UnaryExpression \| “**!**” UnaryExpression |
| PrimaryExpression | ::= | Var \| Literal \| “**(**“ Expression “**)**” |
| VarOrURI | ::= | Var \| QuotedURI |
| VarOrUriOrLiteral | ::= | Var \| QuotedURI \| Literal |
| Var | ::= | “**?**” Identifier |
| Identifier | ::= | <IDENTIFIER> |
| URI | ::= | <URI> |
| QuotedURI | ::= | “**<**” <URI> “**>**” |
| Literal | ::= | URI \| NumericLiteral \| TextLiteral \| BooleanLiteral \| NullLiteral |
| NumericLiteral | ::= | <INTEGER\_LITERAL> \| <FLOATING\_POINT\_LITERAL> |
| TextLiteral | ::= | <STRING\_LITERAL> (“**@**” Identifier)? (“**^^**” URI)? |
| BooleanLiteral | ::= | <BOOLEAN\_LITERAL> |
| NullLiteral | ::= | <NULL\_LITERAL> |
